# Sporadic Fibrodysplasia Ossificans Progressiva in an Egyptian Infant with c.617G > A Mutation in *ACVR1* Gene: A Case Report and Review of Literature

**DOI:** 10.1155/2013/834605

**Published:** 2013-01-23

**Authors:** Mohammad Al-Haggar, Nermin Ahmad, Sohier Yahia, Amany Shams, Bothina Hasaneen, Rasha Hassan Hassan, Yahya Wahba, Nanees Abdel-Badie Salem, Dina Abdel-Hady

**Affiliations:** ^1^Genetics Unit, Pediatrics Department, Faculty of Medicine, Mansoura University, P.O. Box 35516, Mansoura, Egypt; ^2^Radiology Department, Mansoura University Children's Hospital, P.O. Box 35516, Mansoura, Egypt; ^3^Department of Anatomy and Embryology, Faculty of Medicine, Mansoura University, P.O. Box 35516, Mansoura, Egypt

## Abstract

Fibrodysplasia ossificans progressiva (FOP) is an autosomal dominant severe musculoskeletal disease characterized by extensive new bone formation within soft connective tissues and unique skeletal malformations of the big toes which represent a birth hallmark for the disease. Most of the isolated classic cases of FOP showed heterozygous mutation in the *ACVR1* gene on chromosome 2q23 that encodes a bone morphogenetic protein BMP (ALK2). The most common mutation is (c.617G > A) leading to the amino acid substitution of arginine by histidine (p.Arg206His). We currently report on an Egyptian infant with a sporadic classic FOP in whom c.617G > A mutation had been documented. The patient presented with the unique congenital malformation of big toe and radiological evidence of heterotopic ossification in the back muscles. The triggering trauma was related to the infant's head, however; neither neck region nor sites of routine intramuscular vaccination given during the first year showed any ossifications. Characterization of the big toe malformation is detailed to serve as an early diagnostic marker for this rare disabling disease.

## 1. Introduction

Fibrodysplasia ossificans progressiva (FOP [MIM 135100]) that occurs in a prevalence of one case every two millions is an autosomal dominant disorder of the connective tissue characterized by progressive disability due to restriction of joints and heterotopic ossification of skeletal muscles rendering body movements impossible. Big toes abnormality is the congenital hallmark of classic FOP. Occasional features include short thumbs, fifth finger clinodactyly, malformed cervical vertebrae, short broad femoral necks, deafness, scalp baldness, cardiac conduction defect, and mild mental retardation [1].

Clinically, the average age of onset of FOP is five years, ranging between birth and 25 years, with 80% of patients showing some heterotopic ossification by the age of 7 years. By the age of 15 years, 95% of patients have severely restricted mobility of the upper limbs [2]. More than 95% of children with FOP are born with characteristic congenitally malformed big toes. Hence the congenital malformation along with the heterotopic calcification is quite sufficient for the clinical diagnosis of FOP [3]. Definition of the unique congenital malformation of big toe in FOP is crucial to serve as a strong indicator for early diagnosis; it includes clinically the shortened big toe with valgus deformity, and radiologically one or more of the following: (1) shortened and sharpened proximal phalanx, (2) hallux valgus angle (HVA) ≥20°, and (3) intermetatarsal angle (IMA) ≥10° [4]. HVA is the angle between the line from center of the first metatarsal base to the center of the first metatarsal head and line connecting the midpoints of proximal and distal articular surfaces of the proximal phalanx, while intermetatarsal angle (IMA) is the angle between line of the first metatarsal bone and line bisecting the diaphyseal portions of the second metatarsal bone [5].

Ossification usually starts in the neck, spine, and shoulder girdle, confined only to skeletal muscles, but spares tongue, smooth muscles of larynx, diaphragm and sphincters, anterior abdominal, cardiac, and extraocular muscles [6]. The patient presents with episodes (flare-ups) of soft tissue swellings which may become painful; these episodes are triggered by trauma, for example, injections, falls, and viral illnesses. The newly formed bones are independent of the normal skeleton and can fuse with it [7]. Over time these swellings might progress distally, amplifying the risk for the patient of being confined to a wheel chair as it cannot be stopped [8].

Involvement of muscles of mastication and even jaw fixation results in feeding difficulties and malnutrition [9]. Extensive involvement of the muscles of the chest wall leads to a restrictive lung disease, pneumonia, and death [10].

Diagnosis is usually based on history and clinical involvement. Surgical excision is followed by recurrence, and therefore biopsies should be avoided. Radiographs are normal early in the course of illness. CT scan is sensitive to detect calcifications. Contrast enhancement CT and MRI can detect preosseous lesions of FOP and help in early diagnosis [11]. Although FOP is easy to detect clinically, yet misdiagnosis and delayed diagnosis are common. Possible differential diagnoses include isolated congenital malformations, brachydactyly, juvenile bone unions, sarcoma, lymphoma, desmoid tumor, and aggressive juvenile fibromatosis [12].

Histopathologically, the FOP lesions start with a brief inflammatory stage containing an intense perivascular B-cell and T-cell lymphocytic infiltrates with subsequent migration of mononuclear inflammatory cells followed by widespread myonecrosis. Then, an intense fibroproliferative reaction associated with robust angiogenesis and neovascularity is noted [13]. As the lesion matures, fibroproliferative tissue undergoes an avascular condensation into cartilage followed by a revascularization stage and osteogenesis in a characteristic process of endochondral ossification. The resultant heterotrophic ossification is normal, histologically mature lamellar bone with marrow elements [14]. All stages of histological development are present in an active FOP lesion, indicating that different regions within the lesion mature at different rates. 

It has been suggested that the primary molecular pathology in FOP patients involves profound dysregulation of the signaling pathway of the bone morphogenetic proteins (BMP) [15–17] which play critical roles in skeletal development by regulating the proliferation, differentiation, and apoptosis of chondrocytes, osteoblasts, and osteoclasts [18]. This suggestion was done because the process of heterotropic bone formation in FOP patients is similar to that induced by BMP implantation in muscle tissue [19].

BMPs, except BMP-1, are members of the transforming growth factor-*β* (TGF-*β*) family [18]. Several members of BMPs are implicated in heterotrophic bone formation in FOP patients, including BMP-2, BMP-4, BMP-5, BMP-6, BMP-7, and BMP-9 [20]. Similar to other members of the TGF-*β* family, the intracellular signal transduction of BMPs is activated by two types of transmembrane serine/threonine kinase receptors: type I (including four types: ALK1, ALK2, BMPR-IA/ALK3, and BMPR-IB/ALK6), and type II (including three types: BMPR-II, ActR-II, and ActR-II B) [21]. Ternary complex containing BMPs, type II receptor, and type I receptor will be formed. In this complex, the type II receptor phosphorylates the type I receptor at the GS domain (glycine and serine residue-rich domain located between transmembrane and kinase domains of type I receptors). Then the activated type I receptor phosphorylates Smad1/5/8 in the cytoplasm which in turn form complexes with Smad4, move into the nucleus, and regulate gene expression [22]. In FOP patients, BMP receptor-Smad axis plays an important role in heterotropic bone formation induced by BMPs [22].

The GS domain is a critical site of all TGF-*β*/BMP type I receptors. It serves as a “molecular switch” for kinase activity within the receptor. Mutations within the asparagines residue in the GS domain will lead to the activation of kinase activity within type I receptor without stimulation by ligand or type II receptor [23]. GS domain is also a binding site of the inhibitory protein FKBP12 that prevents leaky activation of the BMP type I receptor in the absence of ligand [24, 25], and FKBP12 also regulates the abundance of the receptor at the membrane through recruiting Smad7-Smurf1 ubiquitin ligase [26]. In FOP cells, leaky activation of BMP signaling in absence of ligand together with accumulation of BMP type I receptors at the cell membrane is seen suggesting altered FKBP12 interactions with the GS domain, leading to uninhibited type I receptor activity [6].

Several suggestions were made to identify the proteins and genes responsible for FOP among all these related to BMP-signaling pathway. Overexpression of BMP-4, point mutations, and deletion of a gene encoding NOGGIN (a BMP antagonist) were among these suggestions [15, 27, 28]. However, in 2006, a conservative genome-wide linkage analysis was conducted using a subset of five families with unambiguous classic features of FOP. Linkage of FOP to 2q23-24 was identified. This interval contains *ACVR1* gene encoding the BMP type I receptor ALK2. DNA sequencing of the *ACVR1* gene (alternatively, *ALK2* (MIM 102576)) determined that the same heterozygous missense mutation in the GS activation domain (c.617G > A) occurs in all affected individuals examined [29]. The *ACVR1* 617G > A mutation which is present in classic FOP patients causes a single amino acid substitution of arginine (R) to histidine (H) in codon 206 within the GS domain. ALK2 (p.Arg206His) is a genetically activated type I receptor. 

Kaplan and his colleagues studied 112 FOP patients in order to define the classic and atypical FOP phenotypes. They divided the atypical presentation into 2 classes: “FOP-plus,” in which patients had the classic defining features of FOP plus 1 or more atypical features, and “FOP variants,” in which there were major variations in 1 or both of the 2 classic defining features of FOP [30]. The classic form of FOP is usually associated with the recurrent *ACVR1* mutation p.Arg206His while other mutations were identified in patients with atypical FOP. These mutations are scattered between the GS domain and kinase domain. 

Heterozygosity for p.Arg202Ile or p.Gly356Asp mutations in the *ACVR1* gene is associated with relatively mild course of disease [8, 31]. A heterozygous p.Gly328Glu mutation was identified in a FOP patient who was born with severe reduction deformities of all digits [8]. Normal big toe or its mild deformity was seen in two FOP patients with heterozygous p.Arg258Ser mutation [32]. The different reported *ACVR1* gene mutations known to be related to FOP phenotype are listed in Table 1.

The prevention and treatment of heterotropic ossification in FOP are the ultimate goal of FOP research. Complete prevention of the flare-ups may be impossible. While muscle biopsy and surgical removal of the heterotropic ossification are contraindicated, avoiding surgical procedures and local anesthesia whenever possible is mandatory. Hyperextension of the jaw in dental procedures is potentially deleterious and should be avoided [33]. Redirection of activity to less physically interactive play may be helpful [34]. 

Short course of prednisone could be used in extremely early symptomatic treatment of flare-ups that affect major joints, the jaw, or the submandibular area. A brief course of well-monitored narcotic analgesia in addition to the use of NSAIDs, COX-2 inhibitors or glucocorticoids could be used to manage painful flair-ups [34]. However, there is no definitive treatment preventing the development of heterotropic ossifications in FOP till now. Based on the findings about the central role of uninhibited activity of mutant ALK2 receptors in the heterotropic ossification, specific inhibitors of BMP receptors have been developed to block this uninhibited intracellular signaling [22]. A specific small chemical inhibitor of BMP type I receptors called dorsomorphin is found to block the induction of osteoblastic differentiation *in vitro* in myoblasts expressing ALK2 (p.Arg206His) and ALK2 (p.Gly356Asp) [35].

Most cases of FOP arise as a result of a spontaneous new mutation. Genetic transmission is autosomal dominant and can be inherited from either parent. Within a family, inherited FOP can show a variable expression. If a parent has FOP, the chance that a child will inherit FOP is 50% [36].

In the current report we describe a female Egyptian infant with classic sporadic FOP who early complained of relevant musculoskeletal pains in addition to her characteristic big toe malformations. Molecular analysis of *ACVR1* gene revealed the most common missense mutation (c.617G > A).

## 2. Case Presentation

**Figure 1:**
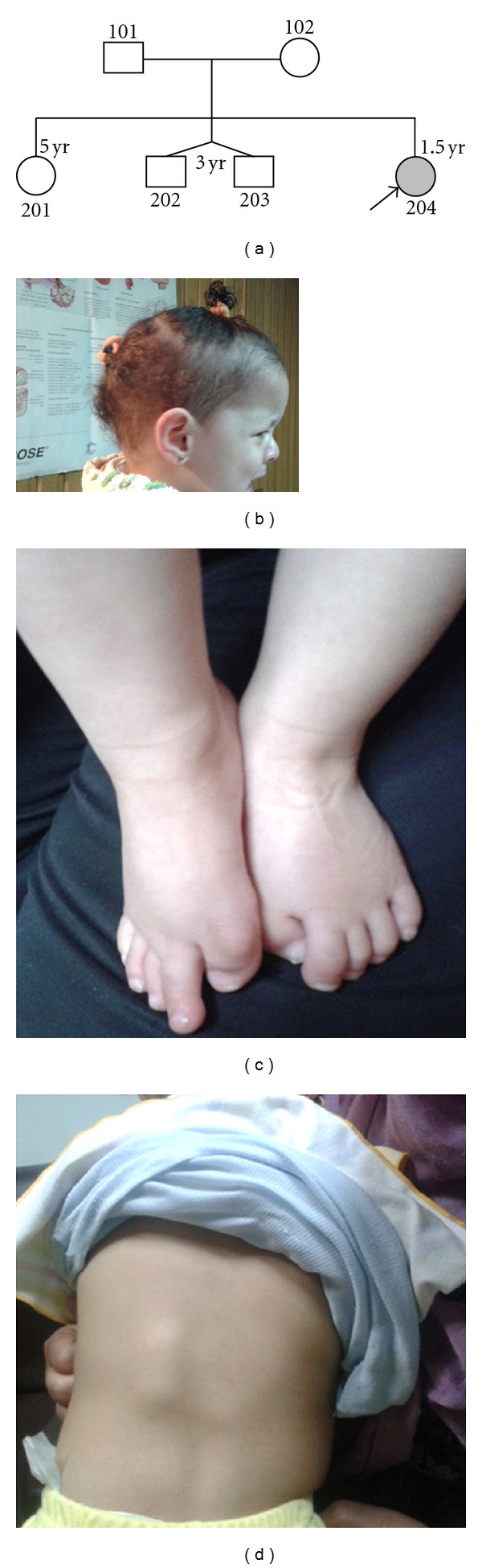
Clinical data: (a) pedigree of the family showing no consanguinity and no family history, (b) photographic profile for the baby's face showing sparse scalp hairs, (c) photograph for the baby's feet showing marked shortening, sharpening of both big toes, with hallux valgus deformities, and (d) para-vertebral hard swellings more on the left side.

An 18-month-old female infant presented with multiple abnormal firm indurations restricted to the back and shoulders. The condition started at the end of the first year following fall down on the head from the bed level. Mother noticed swelling of the face, neck, and shoulder with marked restriction to their movement; it was initially presumed as hematoma although there was no associated skin discoloration or black eyes. With anti-inflammatory therapy (Ibuprofen), pain and swelling regressed leaving only some restricted mobility. At the age of 15 month, multiple painful hard swellings mainly on the upper back had been noticed. Patient sought medical advice and had undergone many radiographic evaluations for the swellings as well as bleeding profile and all were found uneventful. On routine current examination, patient was found average built (height is 79.5 cm, 25–50th centile, weight is 11 Kg, 50th centile, head circumference is 47.5 cm, 75–90th centile). Patient had sparse scalp hair (Figure 1(b)) and was generally well apart from the limited trunk and neck flexibility with clumsy gait and repeated fall down, hard swellings were noticed in the left thoracic cage and back (Figure 1(d)), the old ones were painless and presumed to be present since birth, and the new ones were painful. Systematic examination was irrelevant apart from the marked shortening and sharpening of both big toes with bilateral hallux valgus deformity (Figure 1(c)), and parents stressed that these malformations were present since birth with no family history of similar condition. There was no pallor, jaundice or cyanosis and no history of recent blood transfusion. The child was full term and born to a nonconsanguineous couple (Figure 1(a)) by caesarean section, and developmental milestones were average for age. Patient had received all her vaccines including the intramuscular (IM) injections at the proper ages with no significant local complications at the vaccination sites.

Complete blood counts revealed microcytic, hypochromic anaemia (HB was 8.9 gm/dL) with normal serum calcium, phosphorus, and alkaline phosphatase. Erythrocyte sedimentation rate and C-reactive protein were normal. Bleeding profile including bleeding, clotting, prothrombin, and activated partial thromboplastin times, all were normal. Radiological evaluation was done using plain X-ray, ultrasound, and CT scan. Patient started anti-inflammatory therapy in form of ibuprofen, interleukin 2 modifiers regularly for 3 months, and short courses of steroid therapy on an “on demand basis” for the control of flare-ups and pains with partial improvement.

## 3. Radiological Diagnosis

**Figure 2:**
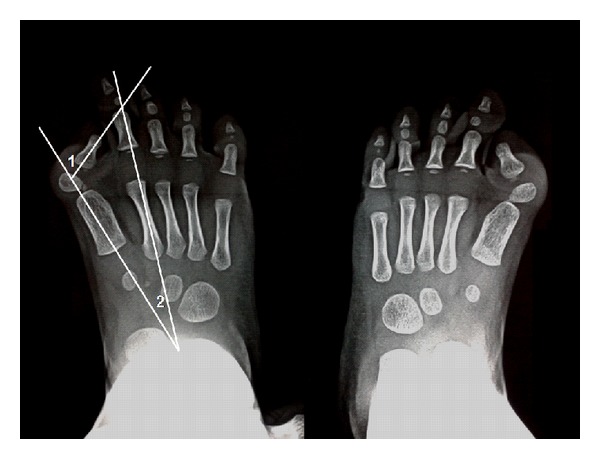
X-ray for the baby's feet showing rudimentary proximal phalanges, hallux valgus deformities with hallux valgus angle (HVA); angle (1) 65° and intermetatarsal angle (IMA); angle (2) 20°.

Skeletal survey at the age of 15 months revealed deformity of the big toe in the form of bilateral hallux valgus, small-sized deformed middle phalanx of the little fingers, and clinodactyly with no abnormal calcifications (Figure 2). Hallux valgus angle (HVA) angle (1) was 65° (normally it should be <15–18°) while intermetatarsal angle (IMA) angle (2) was 20° (normally it should be <10°); hence, both were abnormal.

**Figure 3:**
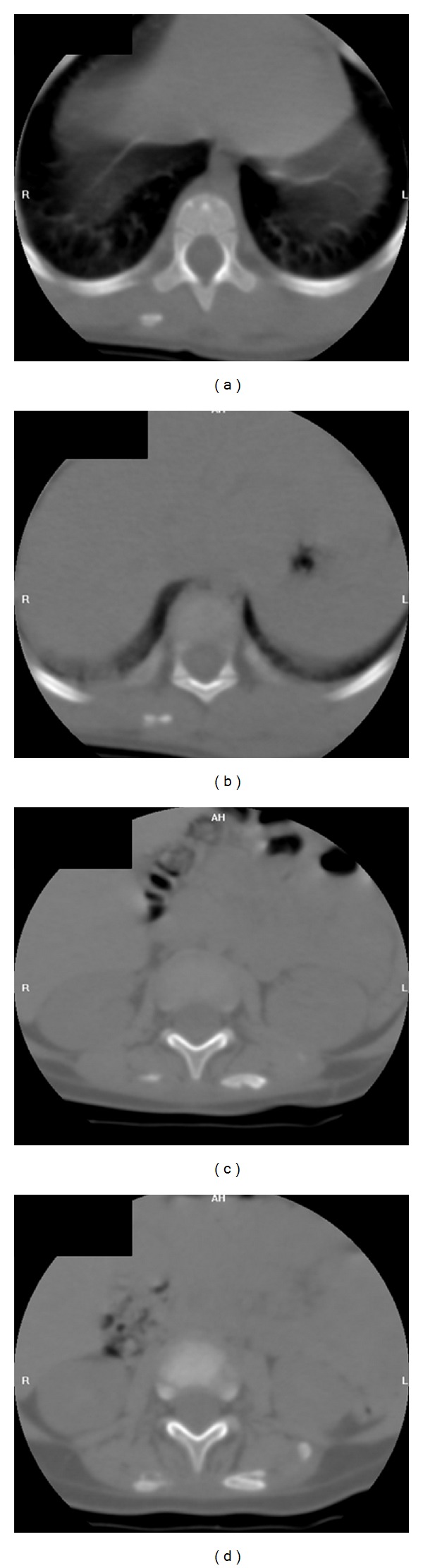
Noncontrast axial CT scan of lumbosacral spine showing multiple plaque-like calcifications in paraspinal muscles, more on the left side, with diffuse muscle thickening.

Ultrasonography for the upper chest and neck done at that age revealed marked thickening of soft tissues at the back of neck and upper chest with swelling of the muscles and intervening soft tissues. Initial pre- and post-contrast CT scan of the neck and upper chest at 15 months confirmed the sonographic results with no initial calcifications, however; the follow-up noncontrast CT at 18 months revealed abnormal thickening of the back muscles with multiple sheet-like calcifications (Figure 3). Diagnosis of FOP was based on the progressive trunk hardening and bilateral marked shortening and inward deviation of both big toes, that is, brachydactyly with pathological hallux valgus malformation.

## 4. Mutation Analysis

**Figure 4:**
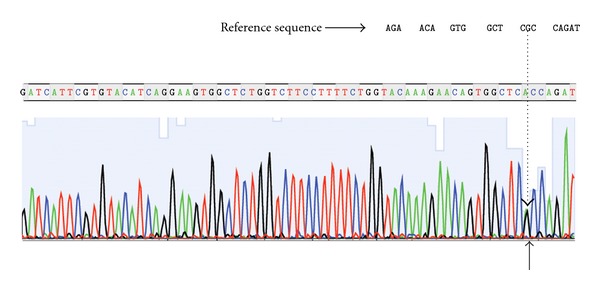
Photograph for exon 4 of *ACVR1* gene for patient showing heterozygous missense mutation at the nucleotide 617 (c.617G > A) leading to the substitution of the amino-acid arginine by histidine (p.Arg206His), note that patient will have the two types of ALK2 proteins (G and A bases appear overlapping at arrow heads).

Molecular testing for *ACVR1* gene was done for all family members in collaboration with the Research Center for Genomic Medicine, Saitama Medical University, 1397-1 Yamane, Hidaka-shi, Saitama, Japan, with the aim to document this early presentation and to provide some phenotype-genotype correlations. Exon 4 of *ACVR1* was amplified using Prime Star HS DNA polymerase (Takara Bio, Inc.) with a set of primers: Sense: CCAGTCCTTCTTCCTTCTTCC, and Anti-sense: AGCAGATTTTCCAAGTTCCATC, in 35 cycles (98°C, 10 sec; 60°C, 5 sec; 70°C, 30 sec). The PCR product was cleaned by Microcon YM-100 (Millipore) and sequenced using Big Dye Terminator v. 3.1 by 3500 Genetic Analyzer (Applied Biosystems, Inc.) [29]. Patient was found heterozygous for c.617G > A mutation in *ACVR1* leading to the amino-acid substitution (p.Arg206His); therefore, the expected amino acids arginine and histidine will be read at the mutation site, and the patient may have two types of ALK2 proteins (Figure 4). All other sibs as well as the nonconsanguineous parents were negative for that mutation.

## 5. Discussion

FOP is a severe disabling musculoskeletal disease characterized by extensive formation of endochondral bone within soft connective tissues and unique skeletal malformations of the big toes that are present at birth and serve a birth hallmark for the disease. Heterotopic bone formation, which is the most clinically relevant feature of FOP, usually starts by the age of five years with 80% before age of 7 [2].

Mutations in the gene encoding *ACVR1*, a BMP type I receptor, had been found in all examined FOP patients, thus confirming that changes in the BMP-signaling pathway that causes the disease process in FOP [29].

Our current Egyptian patient started to complain of musculoskeletal pains following mild trivial head trauma towards the end of the first year of life, swelling was initially vague; it was in the form of hard nonpitting diffuse edema which was not restricted to the area of trauma but extends to involve a wide area around; it involved the face, neck, and upper back. Mutation of *ACVR1* gene c.617G > A (p.Arg206His) described in that case was identical to that found in most classic cases of FOP [29]. This mutation was considered to be the activator that stimulates BMP signaling without requiring BMP to initiate the signaling cascade. Existence of congenital toe malformation and acquisition of heterotopic calcification were explained by the embryonic skeletal morphogenesis theory that when dysregulated, an inadvertent ectopic chondroosseous differentiation of soft connective tissue will be triggered leading to a disabling heterotopic new bone formation [37].

Metamorphosis, which encompasses the transformation of one normal tissue into another, is a biological process rarely studied in higher vertebrates or mammals, but exemplified pathologically by FOP. *ACVR1* gene (ALK2 protein; a bone morphogenetic protein (BMP) type I receptor) is the first identified human metamorphogene; its common missense mutation c.617G > A that substitutes arginine by histidine (p.Arg206His) causes skeletal metamorphosis in all classically affected FOP cases worldwide. Physiological studies of this metamorphogene are beginning to provide deep insight into a highly conserved signaling pathway that regulates tissue stability following morphogenesis, and that, when damaged at a highly specific locus (c.617G > A) and triggered by an inflammatory stimulus permits metamorphosis of normal functioning connective tissue into a highly ramified skeleton of heterotopic bone [6]. Other mutations of ALK2 were identified in FOP patients with milder form or slowly progressive disease. Quantitative difference between activity of ALK2 with these mutations might provide some phenotypic differences in these patients [38]. This dominant single-gene disorder although, manifested at birth by the bilateral symmetrical hallux valgus, triggered by blunt trauma that usually occurs at the age of walking. 

An interesting finding in the current case who had developed many localized inflammatory reactions by the IM injections of vaccines and medications, however, was that there were no clinical or radiologically detectable in situ ossifications. Therefore inflammatory trigger of metamorphosis could be remote rather than local, and the search for orthotopic clacification should be related to the embryonic skeleton rather than to the site of trauma. Clinical diagnosis of FOP should be considered in infants who develop pronounced hard edema after blunt trauma with the lack of strict localization, lack of bruising, or evidences of bleeding tendency especially if associated with congenital hallux valgus malformation.

At present, no treatments are available to prevent heterotopic bone formation in FOP, our patient had been on many anti-inflammatory medications without marked change of the clinical course. Molecules, including dorsomorphin and Smad7, will aid in the establishment of novel methods of treatment of FOP [35]. Establishment of some animal models of FOP could pave the way for development of novel therapeutic modalities for FOP [39].

Our Egyptian patient with classic FOP phenotype showed heterozygosity of p.Arg206His mutation in *ACVR1* gene, a mutation considered to be the commonest and the most recurrent in classic FOP patients worldwide. We can conclude that the congenital hallux valgus malformation should be used as a gold standard for diagnosis with strict search for orthotopic calcification, after trauma exposure, in areas related to embryonic skeleton. The heterozygous p.Arg206His mutation in *ACVR1* gene showed consistency to classic FOP phenotype. 

## Figures and Tables

**Table 1:** Reported *ACVR1 *gene mutations known to be related to FOP phenotype.

Mutation type	Accession number	Codon number	Codon change	Amino acid change
Missense/nonsense	CM091832	202	AGA-ATA	Arg-Ile
Missense/nonsense	CM061633	206	CGC-CAC	Arg-His
Missense/nonsense	CM091417	207	cCAG-GAG	Gln-Glu
Missense/nonsense	CM090905	258	AGGc-AGC	Arg-Ser
Missense/nonsense	CM091418	328	aGGG-AGG	Gly-Arg
Missense/nonsense	CM091419	328	aGGG-CGG	Gly-Arg
Missense/nonsense	CM091420	328	aGGG-TGG	Gly-Trp
Missense/nonsense	CM091421	328	GGG-GAG	Gly-Glu
Missense/nonsense	CM080031	356	GGC-GAC	Gly-Asp
Missense/nonsense	CM091422	375	CGT-CCT	Arg-Pro
Small deletions*	CD091423	TCTGGT^*∧*196^CTTCcttTTCTGGTAC		

Based on HGMD (http://www.hgmd.cf.ac.uk/ac/gene.php?gene=ACVR1) (last visited at 21/12/2012).

*Microdeletions (20 bp or less) are presented in terms of the deleted bases in lower case plus, in upper case, 10 bp DNA sequence flanking both sides of the lesion. The numbered codon is preceded in the given sequence by the caret character (^*∧*^).

## References

[1] Subramanyam L, Gowrishankar K, Shivbalan S, Balachandran A (2004). Fibrodysplasia ossificans progressiva. *Indian Journal of Pediatrics*.

[2] Cohen RB, Hahn GV, Tabas JA (1993). The natural history of heterotopic ossification in patients who have fibrodysplasia ossificans progressiva: a study of forty-four patients. *Journal of Bone and Joint Surgery A*.

[3] Kaplan FS, Glaser DL, Shore EM (2005). The phenotype of fibrodysplasia ossificans progressiva. *Clinical Reviews in Bone and Mineral Metabolism*.

[4] Nakashima Y, Haga N, Kitoh H (2010). Deformity of the great toe in fibrodysplasia ossificans progressiva. *Journal of Orthopaedic Science*.

[5] Schneider W, Csepan R, Knahr K (2003). Reproducibility of the radiographic metatarsophalangeal angle in hallux surgery. *Journal of Bone and Joint Surgery A*.

[6] Kaplan FS, Le Merrer M, Glaser DL (2008). Fibrodysplasia ossificans progressiva. *Best Practice and Research*.

[7] Shore EM, Kaplan FS (2008). Insights from a rare genetic disorder of extra-skeletal bone formation, fibrodysplasia ossificans progressiva (FOP). *Bone*.

[8] Petrie KA, Lee WH, Bullock AN (2009). Novel mutations in ACVR1 result in atypical features in two fibrodysplasia ossificans progressiva patients. *PLoS ONE*.

[9] Marrannes J, Box I, Haspeslagh M, Gryspeerdt S (2006). Jaw fixation as the key to diagnosis of fibrodysplasia ossificans progressiva. *Journal Belge de Radiologie*.

[10] Hagiwara H, Aida N, Machida J, Fujita K, Okuzumi S, Nishimura G (2003). Contrast-enhanced MRI of an early preosseous lesion of fibrodysplasia ossificans progressiva in a 21-month-old boy. *American Journal of Roentgenology*.

[11] Herring JA (2002). Fibrodysplasia ossificans progressive. *Tachdijan'S Paediatric OrthopaedicS*.

[12] Kitterman JA, Kantanie S, Rocke DM, Kaplan FS (2005). Iatrogenic harm caused by diagnostic errors in fibrodysplasia ossificans progressiva. *Pediatrics*.

[13] Gannon FH, Valentine BA, Shore EM, Zasloff MA, Kaplan FS (1998). Acute lymphocytic infiltration in an extremely early lesion of fibrodysplasia ossificans progressiva. *Clinical Orthopaedics and Related Research*.

[14] Pignolo RJ, Suda RK, Kaplan FS (2005). The fibrodysplasia ossificans progressiva lesion. *Clinical Reviews in Bone and Mineral Metabolism*.

[15] Shafritz AB, Shore EM, Gannon FH (1996). Overexpression of an osteogenic morphogen in fibrodysplasia ossificans progressiva. *The New England Journal of Medicine*.

[16] Gannon FH, Kaplan FS, Olmsted E, Finkel GC, Zasloff MA, Shore E (1997). Bone morphogenetic protein 2/4 in early fibromatous lesions of fibrodysplasia ossificans progressiva. *Human Pathology*.

[17] Olmsted EA, Kaplan FS, Shore EM (2003). Bone morphogenetic protein-4 regulation in fibrodysplasia ossificans progressiva. *Clinical Orthopaedics and Related Research*.

[18] Katagiri T, Suda T, Miyazono K, Derynck R, Miyazono K (2008). The bone morphogenetic proteins. *The TGF-B Family*.

[19] Kaplan FS, Tabas JA, Zasloff MA (1990). Fibrodysplasia ossificans progressiva: a clue from the fly?. *Calcified Tissue International*.

[20] Sampath TK, Reddi AH (1983). Homology of bone-inductive proteins from human, monkey, bovine, and rat extracellular matrix. *Proceedings of the National Academy of Sciences of the United States of America*.

[21] Miyazono K, Maeda S, Imamura T (2005). BMP receptor signaling: transcriptional targets, regulation of signals, and signaling cross-talk. *Cytokine and Growth Factor Reviews*.

[22] Katagiri T (2010). Heterotopic bone formation induced by bone morphogenetic protein signaling: fibrodysplasia ossificans progressiva. *Journal of Oral Biosciences*.

[23] Wieser R, Wrana JL, Massague J (1995). GS domain mutations that constitutively activate T*β*R-I, the downstream signaling component in the TGF-*β* receptor complex. *The EMBO Journal*.

[24] Wang T, Li BY, Danielson PD (1996). The immunophilin FKBP12 functions as a common inhibitor of the TGF*β* family type I receptors. *Cell*.

[25] Chen YG, Liu F, Massagué J (1997). Mechanism of TGF-*β* receptor inhibition by FKBP12. *The EMBO Journal*.

[26] Yamaguchi T, Kurisaki A, Yamakawa N, Minakuchi K, Sugino H (2006). FKBP12 functions as an adaptor of the Smad7—Smurf1 complex on activin type I receptor. *Journal of Molecular Endocrinology*.

[27] Xu MQ, Feldman G, Le Merrer M (2000). Linkage exclusion and mutational analysis of the noggin gene in patients with fibrodysplasia ossificans progressiva (FOP). *Clinical Genetics*.

[28] Sémonin O, Fontaine K, Daviaud C, Ayuso C, Lucotte G (2001). Identification of three novel mutations of the noggin gene in patients with fibrodysplasia ossificans progressiva. *American Journal of Medical Genetics*.

[29] Shore EM, Xu M, Feldman GJ, Fenstermacher DA, Brown MA, Kaplan FS (2006). A recurrent mutation in the BMP type I receptor ACVR1 causes inherited and sporadic fibrodysplasia ossificans progressiva. *Nature Genetics*.

[30] Kaplan FS, Xu M, Seemann P (2009). Classic and atypical fibrodysplasia ossificans progressiva (FOP) phenotypes are caused by mutations in the bone morphogenetic protein (BMP) type I receptor ACVR1. *Human Mutation*.

[31] Furuya H, Ikezoe K, Wang L (2008). A unique case of fihrodysplasia ossificans progressiva with an ACVR1 mutation, G356D, other than the common mutation (R206H). *American Journal of Medical Genetics A*.

[32] Bocciardi R, Bordo D, Di Duca M, Di Rocco M, Ravazzolo R (2009). Mutational analysis of the ACVR1 gene in Italian patients affected with fibrodysplasia ossificans progressiva: confirmations and advancements. *European Journal of Human Genetics*.

[33] Urtizberea JA Fibrodysplasia ossificans progressiva (FOP). http://www.orpha.net/data/patho/GB/uk-fop.pdf.

[34] Pignolo RJ, Kaplan FS, Shore EM Pediatric Fibrodysplasia Ossificans Progressiva (Myositis Ossificans) Treatment & Management. http://emedicine.medscape.com/article/1007104-treatment#showall.

[35] Fukuda T, Kohda M, Kanomata K (2009). Constitutively activated ALK2 and increased SMAD1/5 cooperatively induce bone morphogenetic protein signaling in fibrodysplasia ossificans progressiva. *The Journal of Biological Chemistry*.

[36] Pignolo RJ, Shore EM, Kaplan FS (2011). Fibrodysplasia ossificans progressiva: clinical and genetic aspects. *Orphanet Journal of Rare Diseases*.

[37] Shen Q, Little SC, Xu M (2009). The fibrodysplasia ossificans progressiva R206H ACVR1 mutation activates BMP-independent chondrogenesis and zebrafish embryo ventralization. *The Journal of Clinical Investigation*.

[38] Fukuda T, Kanomata K, Nojima J (2008). A unique mutation of ALK2, G356D, found in a patient with fibrodysplasia ossificans progressiva is a moderately activated BMP type I receptor. *Biochemical and Biophysical Research Communications*.

[39] Katagiri T (2010). Genetic basis for skeletal disease. Establishment of novel treatments for fibrodysplasia ossificans progressiva (FOP). *Clinical Calcium*.

